# The Role of Oxidative Stress in Myocardial Ischemia and Reperfusion Injury and Remodeling: Revisited

**DOI:** 10.1155/2016/1656450

**Published:** 2016-05-25

**Authors:** Gino A. Kurian, Rashmi Rajagopal, Srinivasan Vedantham, Mohanraj Rajesh

**Affiliations:** ^1^School of Chemical and Biotechnology, SASTRA University, Thanjavur 613401, India; ^2^Department of Pharmacology & Therapeutics, College of Medicine & Health Sciences, UAE University, P.O. Box 17666, Al Ain, UAE

## Abstract

Oxidative and reductive stress are dual dynamic phases experienced by the cells undergoing adaptation towards endogenous or exogenous noxious stimulus. The former arises due to the imbalance between the reactive oxygen species production and antioxidant defenses, while the latter is due to the aberrant increase in the reducing equivalents. Mitochondrial malfunction is the common denominator arising from the aberrant functioning of the rheostat that maintains the homeostasis between oxidative and reductive stress. Recent experimental evidences suggest that the maladaptation during oxidative stress could play a pivotal role in the pathophysiology of major cardiovascular diseases such as myocardial infraction, atherosclerosis, and diabetic cardiovascular complications. In this review we have discussed the role of oxidative and reductive stress pathways in the pathogenesis of myocardial ischemia/reperfusion injury and diabetic cardiomyopathy (DCM). Furthermore, we have provided impetus for the development of subcellular organelle targeted antioxidant drug therapy for thwarting the deterioration of the failing myocardium in the aforementioned cardiovascular conditions.

## 1. Introduction

Cardiovascular diseases (CVD) generally denote disorders of the heart and blood vessels that include coronary heart disease, cerebrovascular disease, and other vascular conditions, and this accounts for the leading cause of death and disability in the world. Interestingly, four out of five CVD deaths are due to heart attack and stroke [1]. The primary approach in understanding the nature of the disease and improving the treatment is to retrospectively study the molecular and cellular signaling mechanisms. Myocardium experiences oxidative challenge in all forms of heart diseases and the oxidative modified molecules not only act as the determinant in the extent of injury but may be useful in the diagnostic and prognostic measures where they can serve as specific biomarkers. In general, cardiomyocytes possess firm defense mechanisms to counter the oxidative challenge via enzymatic and nonenzymatic molecules [2]. It is generally perceived that molecules which neutralize the free radicals generated in the tissues could be beneficial in ameliorating several pathologies, and hence this notion provides the foundation to develop these molecules as drug candidates in combating oxidative tissue mediated tissue injury.

However, a recent clinical study emphasized the significance and pathogenic consequences of proteotoxicity and proteinopathy in the failing human hearts. A homeostatic balance (proteostasis) between synthesis and degradation of defective proteins is crucial for sustaining the health of dynamically active cardiomyocytes [3]. Hence the accumulation of reducing molecules that resulted in the reductive stress (abnormal increase in reducing equivalents) could lead to dysfunction of the endoplasmic reticulum (an organelle involved in the proteins synthesis and folding) and proteotoxicity [3, 4]. Similarly, reactive oxygen species (ROS), a key player that induces oxidative stress, participate not only in the pathological roles in the heart diseases, but also in the physiological function that may regulate survival and demise of the cardiomyocytes [2]. Myocardial adaptation to oxidative/reductive stress is a crucial mechanism evolved for the survival of heart from different disorders. Herein we have discussed the current understanding regarding the proximal relationship between oxidative stress, reductive stress, and the corresponding cellular adaptation process in the diseased heart, chiefly associated with ischemia/reperfusion injury and diabetic cardiomyopathy (DCM).

## 2. Oxidative Stress in Cardiovascular Diseases

The oxidant and antioxidant imbalance in the cardiomyocytes that favors the accumulation of oxidants, leading to cellular damage, constitutes oxidative stress [5]. Generally cells will initiate an adaptive system to protect them against the dangerous effects of oxidative stress, but when the oxidant concentration often exceeds the cell's adaptive capacity, the cell will experience exacerbated oxidative stress. There exist misnomers when referring to the terms such as oxidative stress and free radical damage, which are often interchanged. The term “oxidative stress” is used to describe the imbalances in redox couples such as reduced to oxidized glutathione (GSH/GSSG) or NADPH/NADP^+^ ratios. While the term “free radical damage” denotes the alterations in the structure and function of the biomolecules such as proteins, lipids, and deoxyribonucleic acid (DNA). The exaggerated generation of reactive free radicals from different metabolites is generally inactivated in the cells by several endogenous antioxidants, and it utilizes the redox couples to regenerate the enzyme or assist in these enzymatic reactions. Thus, the terms “oxidative stress” and “free radical damage” are not synonymous in the precise sense, but their biological effects are interdependent.

**Figure 1:**
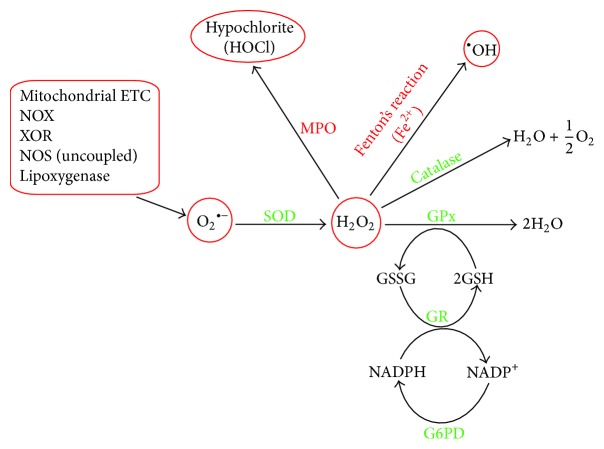
This scheme shows the sources for O_2_^•−^ generation and its detoxification by endogenous antioxidants. O_2_^•−^ is dismutated by SOD resulting in the generation of H_2_O_2_. Then H_2_O_2_ is detoxified via catalase or glutathione peroxidase (GPx) involving GSH. GSSG is recycled with the aid of glutathione reductase (GR). The reducing equivalents are recycled via glucose-6-phosphate dehydrogenase (G6PD). Myeloperoxidase (MPO) utilizes H_2_O_2_ as substrate to produce the powerful oxidant, HOCl, which damages the biomolecules such as lipids, proteins, and nucleic acids. Similarly hydroxyl radical (^•^OH) formed via Fenton's reaction also attacks the biomolecules.

Oxidative stress related to heart was evident in patients undergoing by-pass surgery, where oxidized glutathione (GSSG) accumulation was found to be negatively correlated with the functional recovery of the myocardium [6]. Currently, the ratio of oxidized glutathione (GSSG) to reduced glutathione (GSH) is used as a marker of oxidative stress, considering the fact that GSH, a tripeptide (*γ*-glutamylcysteinylglycine) containing free thiol group, is one of the most important scavengers of ROS in the heart [7, 8]. Furthermore, it can act as an antioxidant in association with glutathione peroxidase (GPx) catalyzed reaction by providing reducing equivalents and thereby helping in the reduction of lipid hydro peroxides (Figure 1). Commonly observed GSH/GSSG ratio in the mammalian heart is relatively high [9], with a 50–70% decrease observed under oxidative stress [10] conditions. It is a general notion that the oxidants are harmful, having no positive or regulatory roles in the biological processes. At low or moderate concentrations, oxidants can act as weapons for the host defense system and this process governs the growth and development of tissues. Thus, tissues have to maintain a tight balance between prooxidant and antioxidant level not only in the organ and tissues, but also in specific cell types. Moreover various cell organelles such as mitochondria, peroxisomes, and endoplasmic reticulum have their own localized antioxidant system. Recently, role of these antioxidant systems is being investigated for understanding the concept of redox signaling and homeostasis towards the pathogenesis of cardiovascular diseases [11].

Oxidative stress is more often associated with elevated levels of ROS or reactive nitrogen species (RNS) in the cellular and subcellular levels [5]. However, ROS/RNS in the suboptimal level can act as signaling molecules in maintaining the cardiovascular function [12]. On the other hand, increased ROS/RNS levels can induce pathology by damaging lipids, proteins, and DNA [13]. Thus, ROS depending on their concentration, the site of production, and the overall redox equilibrium of the cell will determine its biological action (beneficial or deleterious) in the tissues. Cardiovascular pathology associated with oxidative stress is observed in several cardiac diseases like ischemia/reperfusion injury [14, 15], atherosclerosis [16], diabetic cardiomyopathy, and so forth [17].

## 3. ROS/RNS in Myocardial Ischemia Reperfusion Injury

ROS include radicals, such as superoxide anion (O_2_^•−^), hydroxyl radical (^•^OH), hydrogen peroxide (H_2_O_2_), singlet oxygen (O^−^), and hypochlorous acid (HClO). RNS includes nitroxyl anion (NO^−^), nitrosonium cation (NO^+^), higher oxides of nitrogen, peroxynitrite (NO_3_^−^), S-nitrosothiols, and dinitrosyl iron complexes [5]. Peroxynitrite is generated when O_2_^•−^ reacts with NO^•^ and facilitates both oxidizing and nitrating reactions of the biomolecules [18, 19].

According to Wolff the ratio of oxygen delivery to oxygen consumption in the heart is around 1.6 to 1.8 times higher than other tissues, suggesting an excess consumption of oxygen in the myocardium [20]. Molecular oxygen when it accepts an electron will form O_2_^•−^ and this univalent reduction can be mediated by either enzymatic (by the action of NADPH oxidase and xanthine oxidases) [21] or nonenzymatic reactions (with redox active compounds such as semiubiquinone of the mitochondrial electron transport chain) [22]. The resultant O_2_^•−^ is converted to hydrogen peroxide (H_2_O_2_), either by the catalytic action of superoxide dismutase (SOD) or through the spontaneous dismutation [19]. Further interaction of H_2_O_2_ with Fe^2+^ will result in the formation of highly reactive hydroxyl radical (OH^•^) via Fenton's reaction (Figure 1). In addition myeloperoxidase (MPO), an important constituent of leukocytes (neutrophils and macrophages) utilizes H_2_O_2_ as their substrate for the generation of hypochlorite (HOCl) an potent oxidant that attacks biological macromolecules such as lipids, proteins, DNA, and glycoproteins [23].

ROS production and release during myocardial ischemia/reperfusion injury was confirmed by trapping these free radicals using nitrone DMPO [24] and alpha-phenyl N-tert-butyl nitrone spin trap probes [25] and measured by electron spin resonance spectrometer. Numerous preclinical [26, 27] and clinical studies [28] have demonstrated that treatment with antioxidants could render cardioprotection and therefore emphasize the involvement of ROS mediated stress injury in the pathology of myocardial ischemia/reperfusion injury (Table 1).

Among the well-known cellular sources of ROS, mitochondria have emerged as primary source due to their ability to sense the cellular oxygen levels. In fact, in the healthy myocardium, ROS is an unintended byproduct of mitochondrial respiration, where its concentration is tightly controlled to low steady state level by SOD [29]. However, in the ischemic heart, NO^•^, O_2_^•−^, and NO_3_^−^ formation are elevated after reperfusion [30]. The electron leakage from complexes I and III of the electron transport chain (ETC) is primarily responsible for O_2_^•−^ generation in the mitochondria [31]. This in turn damages cardiolipin [32], the phospholipid component of the inner mitochondrial membrane resulting in the destabilization of ETC complexes and supercomplexes, ultimately leading to decreased ATP production and disorganizing the dimeric ADP/ATP carrier functional capacity [33]. Thus, ROS mediated mitochondrial dysfunction and the sequence of biochemical events following revascularization of ischemic area are the cardinal features of the myocardial ischemia/reperfusion injury.

Palmer et al. showed that the heart comprises two spatially distinct mitochondria, namely, interfibrillar (IFM) and subsarcolemmal mitochondria (SSM) [34]. We have demonstrated that IFM and SSM populations respond differently to the oxidative stress induced by myocardial ischemia/reperfusion [35]. This difference in the activities of IFM and SSM towards ROS may be partly due to their spatial location and the metabolic system that detoxifies the radical. It was shown that certain oxidants like H_2_O_2_ are capable of diffusing across the mitochondrial membrane into the cytoplasm [36], while O_2_^•−^ generated in the mitochondria is unlikely to escape into the cytoplasm, indicating the need for localized antioxidant system in the mitochondrial subcompartments. In fact, recent studies have demonstrated that O_2_^•−^ generation was higher in SSM when compared with IFM [37]. In this regard our studies also accede with these observations [38, 39]. Emerging evidences reinforce the concept that ROS could act as regulatory molecules and could protect the myocardium against myocardial ischemia/reperfusion injury [12]. Therefore considering the beneficial effect of ROS, it can be proposed that lower concentration of radicals in IFM during reperfusion phase may possibly activate redox signaling cascade, culminating in the cardioprotection. However, this hypothesis needs to be proven with carefully planned experiments.

## 4. Physiological and Pathological Role of Cardiac Redox Signaling

Redox sensitive modulation of different cardiac proteins such as receptors, transporters, phosphatases, transcription factors like hypoxia inducible factors (HIF), and nuclear factor-*κ*B (NF*κ*B) are well established in the setting of myocardial ischemia/reperfusion injury [40]. It was found that posttranslational modifications of these proteins by ROS/RNS are responsible for the development of CVD [41, 42]. In particular, the Cys-residues in the cardiac proteins undergo oxidative posttranslational modification by the formation of either cysteine thiols, hydroxylation, or nitration which may determine their structure and function [41]. The redox signaling is integral to the maintenance of cardiomyocyte homeostasis. The postnatal cardiomyocyte differentiation and proliferation depends on the redox activation of phosphatidyl inositol 3-kinase, Akt pathway that in turn modulates the *β* catenin signaling, whereby regulating the proper cardiac specification, progenitor expansion, and myocardial growth [43].

Bergmann et al. reported the gradual decrease in cardiomyocyte renewal from 1% at adolescent age to <0.3% in advanced age, indicating the ability of cardiomyocytes regeneration in the adult human hearts [44]. In fact, different studies in lower vertebrates [45], neonatal [46], and adult hearts showed that the main source of cardiomyocyte turnover is from the preexisting cardiomyocytes, rather than from undifferentiated progenitor population [47]. In this direction, it is pertinent to note that thymosin *β*4 (T *β*4), a G-actin ensiling protein involved in plethora of biological functions, has been demonstrated to confer cardiac protection against ischemic insult and promote myocardial regeneration via recruiting HIF-1*α* and suppression of oxidative stress [48]. Moreover, redox signaling is involved not only in the physiological processes and homeostatic pathways, but also in the pathology like fibrosis (adverse cardiac remodeling), where it plays a role in cell metabolism to regulate growth and survival. S-Nitrosylation of the protein kinase B/Akt has been reported to be inactivated during insulin resistance and cardiac dysfunction [49]. Similarly, another study reported that reversible S-nitrosylation of complex I slows the reactivation of mitochondria during the early phase of the reperfusion, thereby reducing ROS production and limiting oxidative tissue damage [50]. Mitochondrial permeability transition pore (MPTP) modulation is associated with electron transport chain derived ROS mediated redox signaling and thereby influences the cellular mitochondrial function as arbitrator or savior of the cell. Perhaps this fact is more significant when considering the point that one of the major constituents of MPTP, namely, the adenine nucleotide translocase, is known to be affected by ONOO^−^ [51]. MPTP is important for the maintenance of mitochondrial structure and function and myocyte differentiation [52]. Thus the redox signaling pathways orchestrated by the mitochondria play a pivotal role in the maintenance of cardiomyocyte structure and function in health and diseases.

Retrograde mitochondrial signaling, a pathway of communication from mitochondria to the nucleus, was reported with hypoxia where the cardiomyocytes gets adapted to the hypoxia through ROS released from mitochondria, resulting in the stabilization of HIF-1*α* and the stimulation of genes responsible for metabolic reprograming towards adapting to the low oxygen tension, and augmentation of collateral circulation via neoangiogenesis [53]. Since myocardium is enriched with mitochondria, ROS emerging from this organelle has been implicated as the key modulator of wide range of cardiomyocyte functions, such as oxygen sensing and mitophagy [54]. Another important physiological role of redox signaling in the myocardium is the regulation of vascular tone by NO^•^ or H_2_O_2_ [55]. H_2_O_2_ plays a key role in vascular function and homeostasis by modifying the protein thiols where it induces cysteine dimerization (R–S–S–R) via the formation of the unstable intermediate sulfenic acid (R–SOH) [55]. Nitric oxide synthase (NOS) isoforms modulate the availability of NO^•^ levels in cells and tissues [56]. Under physiological oxidative stress, NO^•^ mediates S-nitrosylation of critical protein thiols and thus averts them from further oxidative modifications by ROS, thereby rendering cardioprotection [56].

The pathological role of cardiac redox signaling features contractile and energetic dysfunction, arrhythmia, transcriptional changes, and mitochondrial free radical release, leading to abnormal myocardial calcium homeostasis [57]. Oxidant mediated impairment of ryanodine receptor is associated with the activation of PKA/CaMKII, leading to the disruption of calcium homeostasis [58]. Furthermore, ROS derived from NADPH oxidase (NOX) activation in different cardiac pathologies such as ischemia/reperfusion injury activates cell stress-response signaling network that includes p38 mitogen activated protein kinases (MAPK) and c-Jun NH_2_-terminal kinase (JNK) [59]. It is also well established that NOX may have a significant role in stress-induced conditioning such as in ischemic preconditioning, which mediates its cardioprotection by activating prosurvival protein kinases such as Akt and Erk1/2 [60]. The elevated myocardial NO^•^ production during ischemia/reperfusion injury has been associated with ventricular arrhythmia and increased infarct size via modulation of protein-S-nitrosylation [61].

Mitochondrial dysfunction is considered to be the prominent feature of myocardial ischemia/reperfusion injury as this cell organelle is the major contributor of ROS as well as the major target for ROS inflicted damage. During sustained mitochondrial dysfunction, the damaged mitochondria are eliminated by mitophagy. Failure of mitophagy can lead to the persistent loss of calcium homeostasis, excess production of ROS, impaired cellular energetics and ATP production, and culminating in the cell death [61]. Simultaneously, the dysfunctional mitochondria also generate signals to induce stress response such as induction of mitochondrial heat shock proteins to augment mitophagy and promote mitochondrial biogenesis [62].

## 5. Reductive Stress in Cardiovascular Diseases

The current approach to understand the cardiovascular pathology is confined to oxidative stress that may occur as a result of augmented ROS generation and/or reduced production of antioxidants. However, with the reversal of prooxidant to antioxidant status results in the accumulation of reducing equivalents and this will cause reductive stress, a notion initially demonstrated in mice expressing the human mutant *α*B-crystallin [63]. Thus, too much of oxidative radicals or reductive species will disrupt the normal physiological function of cells, which underscores the need to strike a balance between prooxidant and antioxidant concentration for proper cellular function. The concept of reductive stress as a potential contributor to heart failure development [64] and progression is further strengthened by the findings of Zhang and his coworkers [65], wherein they showed the development of cardiomyopathy is more profound in cardiac-specific overexpression of heat shock protein 27 (Hsp27) transgenic mice. The function of Hsp27 is primarily to render cardioprotection via its antioxidative functions. In addition, myocardial ischemia/reperfusion injury is reported to be associated with a hypoxic state that results in an increased NADH/NAD^+^ ratio, leading to a reductive cytosolic environment [66].

The aforementioned phenotypic changes that occur during myocardial ischemia/reperfusion injury could be coupled with mitochondrial dysfunction where GSH-mediated reductive stress in mitochondria is corroborated by decreased expression of redox biosensors, mitochondrial reduction-oxidation proteins, or the oxidation of mitochondrial thioredoxin. Under physiological conditions, GSH has a relatively low redox potential (−240 mV at pH 7.0) and at high intracellular levels (1–13 mm) making it a primary determinant of the cellular redox environment [67]. The pathophysiology of reductive stress varies between cell types as their subcellular compartments have different redox requirements, primarily driven by the reduced (GSH) and oxidized glutathione (GSSG) redox couple [68] and also based on the functional requirement of the organ/tissue. The GSH/GSSG ratio ranges from 30 : 1 to 100 : 1 in the cytosol which results in a redox potential of −290 mV [69]. In an oxidized environment the ratio of redox couple changes in order to achieve a potential difference between −170 and −185 mV. However, in mitochondria, the GSH/GSSG ratios are 20 : 1 to 40 : 1 making the redox potential difference of −250 mV to −280 mV at pH 7.8. Recently, Korge et al. showed that reductive stress can even influence the release of ROS by modulating reduced glutathione reductase (GR) and thioredoxin (Trx), which can donate electrons to O_2_ when the supply of their natural electron acceptors (GSSG for GR and oxidized Trx for Trx) is limited or electron transport to acceptors is inhibited. The above phenotypic changes result in the impairment of ROS-scavenging capacities by GSH/GPx/GR and Prx/Trx/TrxR2 systems [68].

During reductive stress, the electron acceptors in the mitochondria are expected to be reduced and some redox sensitive proteins can donate their electrons to O_2_, leading to ROS production. Similarly, the redox regulatory system includes thioredoxin (Trx) and glutaredoxin (Grx) in the cytosol and mitochondria play an important role in providing reducing equivalent for DNA synthesis, maintaining cellular thiol-redox homeostasis, protection against oxidative stress, governing protein folding, and regulation of cell growth/apoptosis [70]. Alterations in GSH homeostasis affect the cellular redox status by disturbing Trx and Grx balance, which accounts for the possible reductive stress, as evidenced in the reperfused heart [68].

**Figure 2:**
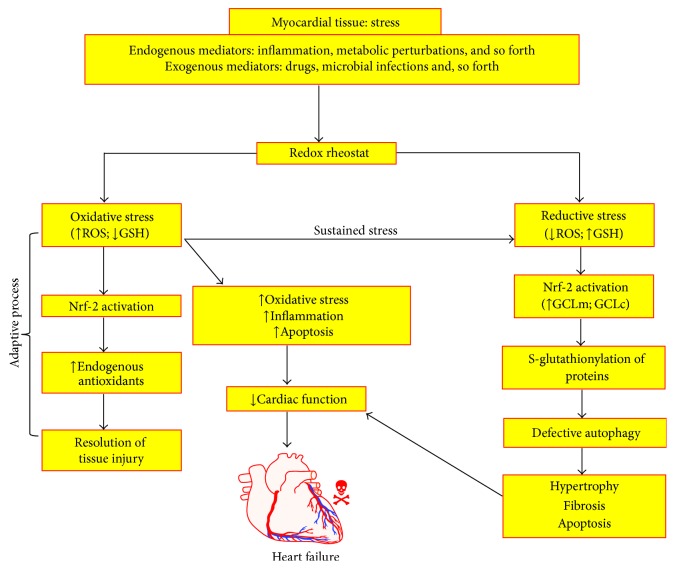
This schematic illustration describes the central role of reductive stress in modulating the myocardial tissue injury. Under normal oxidative stress condition, activation of Nrf-2 results in augmenting endogenous defense system, which aids in the resolution of the tissue injury. However, during sustained oxidative stress condition, Nrf-2 is profoundly activated, which results in the production of increased reducing equivalents such as GSH, which then indulges in posttranslational modification of critical proteins in cardiomyocyte function, whereby affecting their structure and function. These phenotypic events culminate in defective autophagy and drive the cardiomyocytes to become hypertrophic, producing extracellular matrix and committing suicide (apoptosis). All these phenotypic events collectively contribute to the pathogenesis of heart failure. Glutamate-cysteine ligase modifier subunit (GCLm); glutamate-cysteine ligase catalytic subunit (GCLc).

One of the important consequences of impaired equilibrium between prooxidants and antioxidant is the opening of MPTP. MPTP's nonselective permeabilization of the inner mitochondrial membrane commenced by the combined forces comprising calcium overload, adenine nucleotide depletion, and oxidative stress, leading to apoptotic cell death during myocardial ischemia/reperfusion injury [71]. Mitochondrial membrane potential regulated by the redox couple will determine the opening and closing of MPTP. Reductive stress mediated cardiac pathology is linked with functional status of cardiac mitochondria and its redox status. There are limited reports available regarding the status of mitochondrial Trx and GRx in IFM and SSM [72, 73]. Therefore, it is imperative to note that the subcellular concentration of redox couple will not only determine the efficacy of the cellular antioxidant system to counter the ROS attack, but also decide the fate of the mitochondrial function. To date, no evidence is available to suggest the differential redox status existing among IFM and SSM that can be helpful in deciding why IFM is more stable than SSM during ischemia/reperfusion injury. The role of reductive stress in pathophysiology of myocardial dysfunction is illustrated in Figure 2.

## 6. Myocardial Cellular Stress Response and Oxidative Stress

Hearts exhibit a remarkable adaptive response to the physiological and pathological changes in order to maintain contractility. Cardiac dysfunction will occur when the compensatory responses are not sustainable. Under the circumstances of oxidative stress, cells will respond by activating certain signal transduction pathways that promotes their survival and if the cellular strategy failed to accomplish its goal, this will activate cell death signaling pathways. Such cellular prosurvival activities include the production of heat shock proteins, unfolded protein, and DNA damage responses [74]. Previous studies have reported the production of protective heat shock protein during ischemia/reperfusion injury [75] and different conditioning techniques which include preconditioning [76] and postconditioning [77]. One major pathway in cellular death is apoptosis that involves the release of cytochrome* C* from mitochondria that in turn binds to a protein known as Apaf-1 (apoptotic protease activating factor 1) and triggers its oligomerisation and subsequent execution of programmed cell death [2].

Heat shock proteins (Hsp) produced in the low stress level are shown to inhibit the proapoptotic pathway; in particular, Hsp27 binds to cytochrome* C* and prevents its binding to Apaf-1 [78]. Similarly, Hsp90 binds to Apaf-1 and prevents its binding to cytochrome* C*, while Hsp70 prevents oligomerised Apaf-1 from recruiting pro-caspase-9, thereby imparting cardioprotection. The above observations are further supported by another study, wherein cardiac-specific overexpression of Hsp70 conferred cardioprotection against myocardial ischemia/reperfusion injury [79, 80]. Mitochondria is considered to be the hub for cellular redox processes and a number of mitochondrial stress signals that emerge participate in cell-to-cell communication and stimulate cellular adaptations to exogenous or endogenous stimulus. The oxidative stress in the cells is perceived by mitochondria and initiates release of stress signals that lead to membrane depolarization, alterations in adenine nucleotide levels, ROS production, Ca^2+^ fluxes, and permeability transition pore opening [81]. When a perturbation in the mitochondrial oxidative phosphorylation occurs, it results in the enhanced rate of ROS generation. This process not only reduces the cellular energetic process (decreased ATP production), but also results in greater propensity for generation of ROS. Collectively, the above homeostatic mechanisms are crucial in the determination of cardiomyocyte recovery response during myocardial ischemia/reperfusion injury.

## 7. Oxidative/Reductive Stress in Cardiac Remodeling and Diabetic Cardiomyopathy

The hallmark pathological characteristics of diabetic cardiomyopathy (DCM) include myocardial hypertrophy [82] and fibrosis [83]. In addition, in diabetic milieu, the metabolic disturbances result in impaired calcium handling, lipotoxicity (increased production of ceramide, etc.) mitochondrial dysfunction, oxidative stress, and altered insulin sensitivity in the cardiomyocytes [84–86]. Furthermore, in subjects with diabetes, increased accumulation of advanced glycated end products (AGEs) in the plasma/serum is reflective of the secondary effect of myocardial collagen cross linkages leading to myocardial stiffness and impaired cardiac relaxation, indicating myocardial remodeling (fibrosis) [87]. A series of pathological events initiated by ROS in diabetic cardiomyopathy revolves around the mitochondrial dysfunction and its impaired functional activities like irregular calcium handling capacity and defective oxidative phosphorylation [88, 89].

Interestingly, human enzyme Aldose reductase (AR), an aldo-keto reductase involved in the development of diabetic cardiovascular complications, has also been implicated in the myocardial tissue injury during ischemia/reperfusion studies, owing to the enhanced generation of ROS [90–92]. In addition, the ROS catalyzes the oxidation of cysteine residues to sulfenic acid which in turn increases the activity of AR [93]. Recent reports have demonstrated the glucose flux via the polyol pathway which can occur during ischemic condition, even in the absence of diabetes [94, 95]. In fact, other studies also reported the increase in AR and succinate dehydrogenase (SDH) activates in the aged hearts [96, 97]. Surprisingly, subsequent studies suggested that mitochondria could play a pivotal role in ischemia/reperfusion injury by opening the MPTP. This in turn is associated with the fact that AR causes increased oxidative stress and depletes GSH, thereby leading to the intracellular accumulation of H_2_O_2_ and its defective dismutation [98]. Another mechanism which postulates the role of AR in ischemia/reperfusion injury is the decreased phosphorylation of cardiac glycogen synthase kinase-3*β* which impairs normal mitochondrial function as well as the functional recovery of heart during the stressed condition [99]. In addition, overactivation of polyol pathway comprising of AR and SDH has also been shown to impair the function of critical proteins such as sacro/endoplasmic reticulum Ca^2+^-ATPase (SERCA) and ryanodine receptor (RyR), whose major role is involved in the regulation of cardiac contractility. This phenomenon has also been implicated in the pathogenesis of myocardial ischemia/reperfusion injury [100]. Further, it was confirmed that the contribution of polyol pathway to ischemia/reperfusion injury was due to the accumulation of Fe^2+^ that exacerbates oxidative stress, which leads to the increased lipid peroxidation [101]. On the contrary, few studies suggest that the increase in AR activity during ischemia was a result of augmented NO^•^ and protein kinase C (PKC) signaling pathways that plays a cardioprotective role [100, 102]. However, this notion needs to be confirmed with rigorous studies.

## 8. Cardiac Remodeling and Ischemia/Reperfusion Injury

Cardiac remodeling comprises of both gross anatomical changes that alter shape, size, and function and cellular modifications like changes in the gene expression and cellular and interstitial and cytoskeletal reorganization [103]. The above-mentioned features may be described as physiological or pathological, mainly to adapt to the diverse cellular stress conditions. For instance, the initial cardiovascular response in higher altitude will be tachycardia with constant stroke volume, but with a slight increase in blood pressure to adapt to the lower partial pressure of oxygen [104]. Similarly, hypertrophy is considered to be the pathological adaptive responses, especially in myocardial infarction, cardiomyopathy, valvular disease, and ischemia/reperfusion injury. Increasing evidence highlights the role of ROS and RNS in the maladaptation of heart during the pathology, through redox signaling process [105]. At least one-quarter of patients who experienced myocardial infarction may develop cardiac remodeling and subsequent heart failure. As a result of an ischemic insult, the number of cardiomyocytes deceases and the surviving myocytes become elongated or hypertrophied in a compensatory process to maintain stroke volume after the loss of contractile tissue. The thickness of the ventricular wall also increases. Similar to cardiomyocytes, other resident cells of the myocardial tissue, such as fibroblast and endothelial cells are also activated by ischemic insult resulting in increased collagen synthesis and fibrosis, thereby contributing to the myocardial remodeling. In general, progression to heart failure is a determinant of the way in which the ventricles counteract the factors that influence the malfunction. ROS is considered to be a major factor in regulation of myocardial remodeling in a number of ways, such that (A) it can act as a signaling molecule in the development of compensatory hypertrophy [106], (B) it may activate matrix metalloproteases (MMPs) that reconfigure the extracellular matrix [107], and (C) it may account for the loss of myocytes via apoptosis or other cell death mechanisms.

Myocardial infarcted heart exhibits ventricular remodeling and the whole process is divided into an early phase (within 72 hours) and a late phase (beyond 72 hours). Left ventricular remodeling is characterized by reorganization of the extracellular matrix, disfiguration of the geometry, interstitial inflammation, fibrosis, extensive ventricular dilatation, and deterioration in cardiac function, resulting in progressive heart failure [108]. In fact, recent studies have postulated that peroxynitrite could protect against myocardial ischemia/reperfusion injury [109, 110]. However this discrepancy needs to be clarified with further experiments. Nonetheless, several studies have suggested that various small molecules conferred cardioprotection against myocardial ischemia/reperfusion injury via mitigating ROS generation, left ventricular hypertrophy, and myocardial fibrosis (Table 2) [111]. However, these contradictory observations involving the role of free radicals in cardiac remodeling provide sufficient evidence for its both beneficial and pathological roles in the development of cardiovascular diseases.

**Figure 3:**
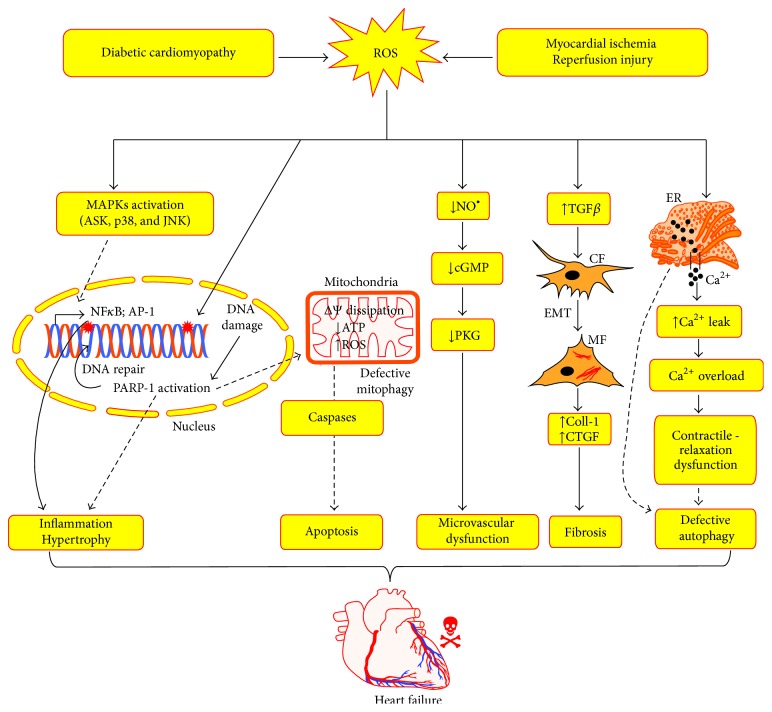
This scheme depicts the role of ROS in the pathophysiology of diabetic cardiomyopathy and myocardial ischemia/reperfusion injury. ROS triggers the activation of MAPKs in the cardiomyocytes, which results in the activation of transcription factors such as NF*κ*B, AP-1. This results in the expression of proinflammatory cytokines and prohypertrophy proteins. Further, ROS directly induces DNA damage and activates poly (ADP-ribose) polymerase (PARP-1) in the nucleus, whereby it mends the damaged DNA. Overactivation of PARP-1 results in depletion of ATP, MPTP opening, mitochondrial dysfunction, and initiation of apoptotic cell death pathways. Next, ROS depletes NO^•^ in the cardiac microvasculatures and promotes endothelial dysfunction via ONOO^−^ generation. Further ROS induces myocardial fibrosis via activation of profibrotic mediators such as TGF*β* and priming the epithelial mesenchymal transition (EMT) process of cardiac fibroblasts (CF) differentiation to myofibroblasts (MF), which produces the extracellular matrix. In addition, ROS also perturbs the calcium handling capacity of the cardiomyocytes and interferes in the autophagy process. All these phenotypic events modulated by ROS orchestrates in the development of cardiac failure. Collagen-1 (Coll-1); connective tissue growth factor (CTGF); cyclic GMP (cGMP); protein kinase G (PKG).

Cellular and systemic response to myocardial ischemia/reperfusion with respect to NO^•^ is considered to be modulatory in nature, as pathological flux may overcome the protective role of NO^•^ [112, 113]. Excess NO^•^ level in the myocardium could be detrimental if it combines O_2_^•−^ to form peroxynitrite radical that can initiate the formation of several other reactive free radicals and aid in myocardial tissue injury [113]. Moreover, peroxynitrite radical can alter the protein function by forming S-nitrosylation and S-glutathiolation, when it combines with sulfhydryl group containing molecules [114, 115]. S-Nitrosylation of cysteine residues as posttranslational modification influence cardiac function includes receptors, enzymes, ion channels, transcription factors, and structural proteins [116]. All these studies discussed herein reveal the fact that a rigid regulatory mechanism is vital in keeping a check on oxidative stress adaptive processes and this could be an apparent prerequisite for a normal cardiac function. In this line of observations, several studies suggest that delineating the molecular mechanisms purported to reverse the cardiac remodeling might serve as tenable therapeutic target in the management of DCM and ischemia/reperfusion injury [117]. The central role of ROS in the pathogenesis of DCM and ischemia/reperfusion injury is schematically illustrated in Figure 3.

## 9. Failure of Antioxidant Based Clinical Trials

Several preclinical studies have indicated the potential cardioprotective actions of antioxidants. However, human clinical studies have failed to establish the cardioprotective activity of antioxidant treatments in reducing infarct size. Further, it did not decrease the risk of mortality rates or retarded the deteriorating myocardial function [118]. This discrepancy perhaps could be due to number of factors that contribute to the failure of clinical trials such as (I) inadequate knowledge of antioxidant pharmacological actions in clinical subjects, (II) insufficient dose response studies, (III) the presence of interfering drugs which could affect the pharmacokinetics of antioxidants, and finally (V) the lack of bonafide biomarkers and clinical end points to evaluate the efficacy of antioxidants against cardiovascular diseases. Most importantly, poor sample size and lack of reproducible studies in different populations across the world impedes our knowledge in unraveling the definitive outcome of antioxidants treatment efficacy in combating cardiovascular diseases.

In spite of these limitations, still the prevalent opinion is that antioxidants can in part delay the inevitable rather than completely preventing the occurrence of myocardial infarction. Despite these setbacks, the focus has now shifted towards targeting mitochondria with the selective antioxidants, since this subcellular organelle is unequivocally involved in the pathophysiology of cardiovascular diseases. In this direction, preclinical studies have strongly shown the proof of concept and evidence that mitochondria targeting antioxidants are in fact effective in preventing the deleterious effects of myocardial ischemia/reperfusion injury and in other rodent models of cardiovascular diseases [119, 120]. However, we need to ascertain if these approaches are successful in the human clinical trials.

## 10. Conclusion

There is a general notion that ROS are always deleterious and hence it should be scavenged. Nevertheless, this is not the case in all the scenarios. A homeostatic balance (proteostasis) between synthesis and degradation of defective proteins is crucial to maintain proper health of the dynamically and metabolically active cardiomyocytes. This balance depends not only on oxidative stress but also with reductive stress in the myocardium and subsequent posttranslational modification of vital and sensitive cardiac proteins that are involved in the basic function of contractile myocytes. Hence the drugs that need to be developed to treat the cardiovascular pathologies like ischemia/reperfusion injury and DCM should be able to modulate both oxidative and reductive stress pathways. Nevertheless, a series of mitochondria targeted antioxidants developed recently provides further impetus to delve further to characterize their pharmacokinetics and pharmacodynamic properties and progress with the pharmaceutical development. In this direction, if we achieve the fruition in clinical trials, these agents would set a milestone in the drug development and establish a new paradigm in the treatment or management of debilitating cardiovascular diseases.

## Figures and Tables

**Table 1:** Clinical trials for evaluating the efficacy of antioxidant based pharmacotherapy in preventing the oxidative stress mediated myocardial tissue damage in cardiovascular diseases.

Drug	Number of subjects	Trial type	Key findings	Reference
N-acetylcysteine (NAC)	98	Double-blind, randomized clinical trial	NAC prevented early remodeling by reducing the level of MMP-2 and MMP-9	[121]
	52	Randomized clinical trial	NAC decreased pump-induced oxidative stress during cardiopulmonary bypass	[28]

Resveratrol	75	Triple-blinded, randomized, parallel, dose-response, and placebo-controlled trial	Resveratrol-rich grape supplement improved the inflammatory and fibrinolytic status in patients who were on statins for primary prevention of CVD	[122]

Rapeseeds	59	Randomized, double-blind, controlled, and crossover study	Intake of a stabilized rapeseed oil enriched in cardioprotective micronutrients prevented the risk of cardiovascular diseases by improving the cholesterol profile and reducing LDL oxidation	[123]

Flavonoids-epicatechin and quercetin	37	Randomized, double-blind, placebo-controlled, and crossover trial	Epicatechin contributed to the cardioprotective effects of cocoa and tea by improving insulin resistance	[124]

Pravastatin	10	Randomized clinical trial	Oral pravastatin reloading before nonemergent coronary artery bypass grafting (CABG) significantly attenuated postoperative inflammation and systemic NO/iNOS concentrations and reduced the myocardial injury	[125]

Magnesium	52	Randomized clinical trial	The extensive treatment of the patients with magnesium influences the cellular response to ischemia and thus induces cardioprotection against oxidative stress	[126]

Coenzyme Q_10_	51	Randomized clinical trial	Coenzyme Q_10_ supplementation at 300 mg/day significantly enhances antioxidant enzymes activities and lowers inflammation in patients who have coronary artery disease during statin therapy	[127]

Silymarin	102	Randomized trial	The anti-inflammatory and antioxidant effects of silymarin treatment provided protection against reperfusion injury and inflammation after CABG surgery	[128]

**Table 2:** Evidence for the amelioration of left ventricle (LV) remodeling by dietary antioxidants and other drugs in preclinical studies.

Antioxidant	Principal findings	Reference
Celiprolol	The *β*-1 blocker at 100 mg/kg that prevented hypoxia induced LV remodeling is in mice, by increasing eNOS	[129]

Fluvastatin	20 mg/kg reduced infarct size and improved the hemodynamics in a rat model of MI	[130]

Pranidipine	The Ca^2+^ channel antagonist at 3 mg/kg improved systolic and diastolic function accompanied by suppressed abnormal gene expression after MI in rats	[131]

Hydrogen sulfide	Exerts antioxidant effects on left ventricular remodeling in rat model of passive smoking via PI3K/Akt-dependent activation of Nrf2 signaling	[132]

Captopril	In patients with anterior MI, it improved left ventricular remodeling and prevented its enlargement, better than digitalis	[133]

Indacaterol + metoprolol	Indacaterol, a new ultra-long-acting *β*2-adrenoceptor agonist at 0.3 mg/kg reversed cardiac remodeling and its effects in combination with metoprolol 100 mg/kg, a selective *β*1-adrenoceptor antagonist in a rat model of heart failure, by reducing cAMP and cardiac GPCR kinase-2 expression	[134]

Vildagliptin	In type 2 DM rats subjected to MI, at 10 mg/kg, the DPP-4 inhibitor restored the autophagy in noninfarcted region and increased survival rate	[135]

Sinapic acid (SA)	SA protected cardiomyocytes and perfused heart from revascularization injury induced oxidative stress by increasing eNOS expression	[136]
